# Cashew Apple Pomace: Chemical Composition and Applications in Functional Food Product Development—A Review

**DOI:** 10.1002/fsn3.70185

**Published:** 2025-04-21

**Authors:** Emmanuel Duah Osei, Anthony Amotoe‐Bondzie, Abigail Ataa Pokuah, Wisdom Sambian Laar, Newlove Akowuah Afoakwah, Eva Ivanišová

**Affiliations:** ^1^ Faculty of Agrobiology, Food, and Natural Resources Czech University of Life Sciences Prague Czech Republic; ^2^ Institute of Food Science, Faculty of Biotechnology and Food Sciences Slovak University of Agriculture Nitra Slovakia; ^3^ Department of Food Science and Technology, Faculty of Agriculture, Food and Consumer Sciences, Nyankpala Campus University for Development Studies Tamale Ghana

**Keywords:** bioactive compounds, cashew apple pomace powder, functional foods, nutritional, valorization

## Abstract

Cashew nut production and fruit processing generate significant by‐products, particularly cashew apple and cashew apple pomace (CAP), which are often treated as waste. However, CAP is a valuable source of nutrients and bioactive compounds that can be repurposed to develop functional food products. Valorizing this by‐product represents a pivotal advancement toward achieving sustainability and circularity in the food industry. This review aimed to highlight the chemical composition and potential applications of CAP in functional food development. The review shows that CAP is enriched with bioactive compounds, including phenolic acids, carotenoids, and flavonoids, alongside essential nutrients such as fiber, minerals, carbohydrates, and proteins. The incorporation of CAP into food products may confer a myriad of health benefits, including antioxidant, antimicrobial, antidiabetic, antiobesity, and gastroprotective properties. This review elucidates approaches to effectively integrate CAP into food formulations while preserving their sensory attributes. Utilizing CAP in food products can significantly reduce food waste and enhance the overall nutritional and functional profile of food, contributing to a more sustainable and circular food system.

## Graphical abstract

Cashew apple pomace (CAP), a nutrient‐rich byproduct of cashew processing, contains bioactive compounds like polyphenols, carotenoids, and nutrients such as proteins, fibre, minerals, and carbohydrates. It exhibits various health benefits, including antioxidant, antimicrobial, antidiabetic, antiobesity, and anti‐inflammatory properties. Additionally, its incorporation into food product development enhances nutritional value and bioactivity while reducing food waste.

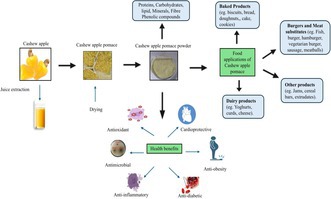

## 1 Introduction

The common cashew ( *Anacardium occidentale* L.) is widely cultivated as an edible nut and cash crop. Its edible portion comprises the cashew nut and the cashew apple (CA), a swollen peduncle often referred to as a pseudofruit (Kolliesuah et al. 2020). Cashew trees are primarily cultivated for their nuts (Madinatou et al. 2023). The CA typically weighs 9–10 times (Esparza et al. 2020) more than nuts, equating to nearly 46 million tons of CA (Akyereko et al. 2022). This pseudofruit is significantly underutilized and often regarded as a waste product in many cases (Tamiello‐Rosa et al. 2019). Around 80% of CA peduncles are discarded yearly (Biasoto et al. 2015).

The perishable nature of CA hinders its effective use in the food industry (de Brito et al. 2018). Drying and pulverizing CA into powder form enables prolonged storage while eliminating its acidity and unpleasant odour. Additionally, this process significantly reduces the antinutritional factors in the resulting CAP (Nagaraja et al. 2011).

The production of CA beverages generates CAP as a by‐product, which is primarily composed of cellulose and hemicellulose. The polysaccharides in this by‐product are subjected to microbial treatment, yielding sugars that are then used to produce various products, including organic acids, bioethanol, and enzymes (Jeyavishnu et al. 2021). While CAP serves as a nutritional ingredient for animal diets in certain regions, it often ends up being discarded and treated as waste, posing environmental concerns (Esparza et al. 2020). The CAP, which makes up approximately 20% of the CA's weight, holds considerable potential for valorization (Parfitt et al. 2010; Rocha et al. 2009).

Establishing recovery routes is crucial to enhance the value of this biomass, which is typically disposed of, burned, or even used as animal feed (Sharma et al. 2020). Each day, individuals require food that provides adequate energy and essential components such as carbohydrates, proteins, fats, vitamins, minerals, and bioactive compounds (Stipanuk and Caudill 2018). CAP offers valuable nutrients, highlighting its potential in response to the rising demand for fibre‐rich, antioxidant foods, and healthy lifestyles (Bianchi et al. 2021).

CAP is known to contain phenolic compounds with potent antioxidant activities and a variety of nutrients such as essential dietary fiber, proteins, minerals, and other health‐enhancing compounds (Preethi et al. 2021; Reina et al. 2022). This by‐product opens possibilities for the food industry to design functional food products. This review focuses on the chemical composition and potential applications of CAP in the development of functional foods.

## 2 Global Production and Botanical Characteristics of Cashew

*Anacardium occidentale* L., commonly known as the cashew tree, is a perennial plant belonging to the Anacardiaceae family. Originally native to Brazil, it was introduced to India and Africa by Portuguese traders in the 16th century for soil erosion control (Tola and Mazengia 2019). Today, cashew cultivation spans approximately 46 countries across Asia, Africa, Latin America, and the Caribbean, covering about 68.56 million hectares and yielding 38.52 million tons of nuts annually, with an average production of 561.9 kg/ha (UNCTAD 2021). Major producers include India, Vietnam, Brazil, and several West African nations, notably Ivory Coast, Nigeria, and Ghana. The global cashew market, previously limited to its native regions, has grown significantly, with production for the 2022/2023 season estimated at 4.6 million tons and a projected market value of $7 billion by 2025, expanding at a rate of 4.3% (Madinatou et al. 2023).

**FIGURE 1:**
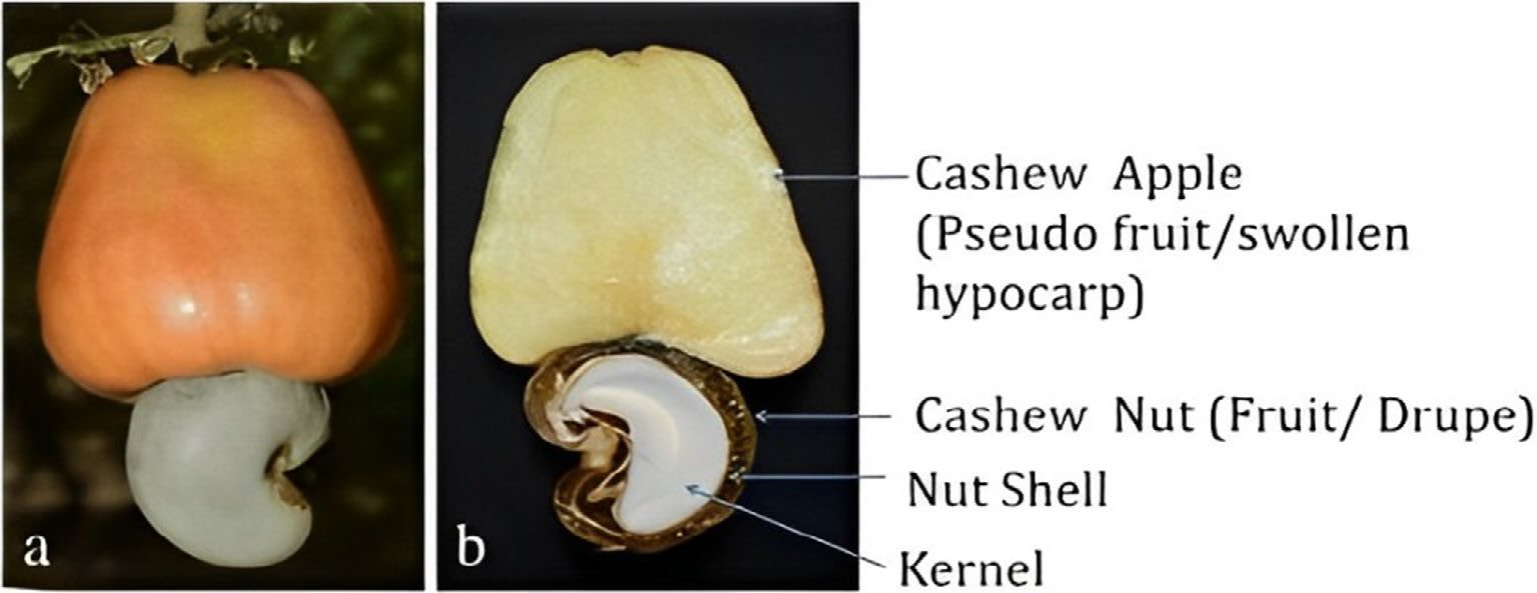
Vertical dissection of a CA with nut (a) reveals that the kernel is encased in the shell (b). The cashew nut is the actual fruit, a drupe, while the CA is a fleshy hypertrophied peduncle or pseudofruit (Savadi et al. 2020).

The cashew apple (CA), a fibrous, juicy fruit with a sour flavour, varies in colour from red to yellow and is typically 8–10 times larger than the nut (Savadi et al. 2020; Singh et al. 2019). The cashew nut structure consists of a kernel, enclosed within a thin layer known as the testa and a thicker outer shell (Figure 1). This shell contains an acidic oil termed cashew nut shell liquid, a byproduct valued for its diverse utility (UNCTAD 2021).

## 3 Cashew Apple Pomace

**FIGURE 2:**
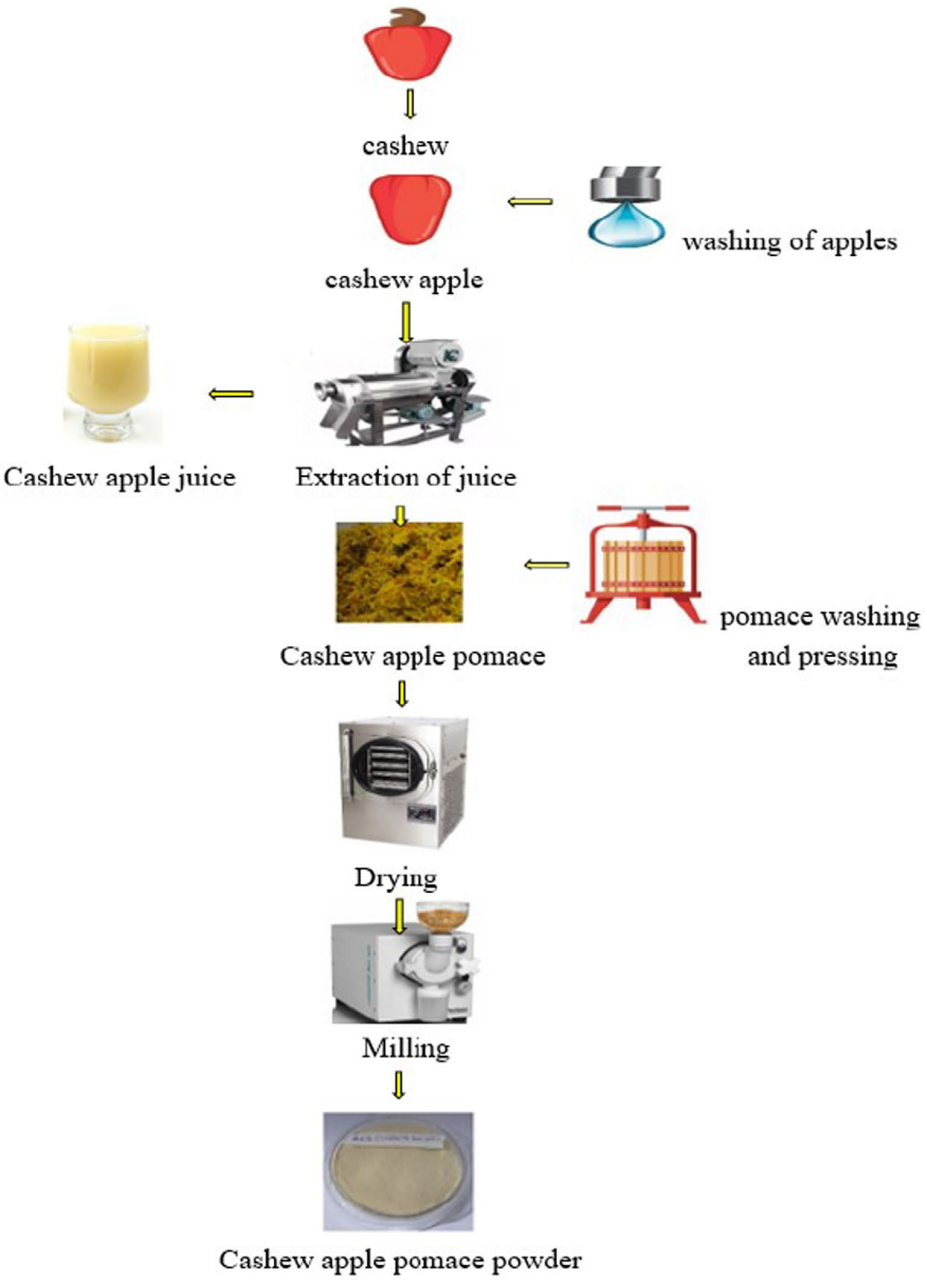
Flowchart for cashew apple pomace powder (CAPP) processing.

The primary by‐product generated during cashew juice processing is CAP, which constitutes lignocellulosic biomass composed of the residual material from the CA processing (Silva et al. 2018). The CAP is generated as waste comprising roughly 40% of the total agroindustrial waste from cashew production (Fonteles et al. 2017). In cashew juice processing, nuts are separated from the fruits by hand or mechanically. The fruits are subsequently cleaned and juiced. The resulting residue, referred to as the pomace and typically regarded as waste, is pressed to extract any remaining juice. As shown in Figure 2, the pressed pomace is then dried to a desirable moisture content, milled into powder, and stored for various food applications.

## 4 Chemical Composition of Cashew Apple Pomace

Studies have reported a myriad of nutrients, including vitamins, minerals, and other secondary metabolites like phenolic and aromatic compounds with bioactive potential (Ndiaye et al. 2022; Reina et al. 2022). In its fresh form, CAP has a high moisture content, ranging from 58%–78.8% (Nurerk and Junden 2021; Patra et al. 2021), which limits its shelf life and food applications. Therefore, drying technologies are essential to achieve a stable, low‐moisture pomace or powder, making it suitable for various food products, particularly in the bakery and confectionery industries.

As shown in Table 1, CAP has been reported to contain up to 22.66% protein, making this edible food by‐product a source of protein for diets, especially in the sub‐Saharan African region, where protein–energy malnutrition remains a common diet‐related problem (Haidar et al. 2003). On a dry basis, CAP has a carbohydrate content of about 26.79%–77.5% (Andrade et al. 2015; donné Kiatti et al. 2024; Reina et al. 2022), lipid content (0.86%–12%), and 78.83 kcal of energy per 100 g (Nurerk and Junden 2021). This suggests that CAP may be used in managing protein–energy malnutrition, as there is a substantial quantity of carbohydrates from which energy can be derived to spare the protein for its primary function (Godswill 2019).

**TABLE 1:** Chemical composition of wet and dry CAP.

Composition	Wet CAP	Dried CAP	Unit	References
Moisture	58–78.8	3.3–9.3	%	Sancho et al. (2015), Guedes‐Oliveira et al. (2016), Kouassi et al. (2018), Silva et al. (2018), Dimoso et al. (2020), de Medeiros et al. (2020), Nurerk and Junden (2021) and Patra et al. (2021)
Carbohydrates	13.9–19	26.79–77.5	%	Nagaraja et al. (2007), Akubor et al. (2014), Duarte et al. (2017), de Medeiros et al. (2020), Nurerk and Junden (2021) and Osei et al. (2024)
Cellulose	18.90	18.24–29.6	mg/100 mL	de Barros et al. (2017), dos Santos Lima et al. (2012) and Rocha et al. (2014)
Hemicellulose	2.6–19.33	27.17–27.19	mg/100 mL	de Barros et al. (2017), dos Santos Lima et al. (2012) and Rocha et al. (2014)
Protein	2.75–16.7	1.83–22.66	%	Podrigues et al. (2007), Guedes‐Oliveira et al. (2016), Duarte et al. (2017), Kouassi et al. (2018), Singh et al. (2019), de Medeiros et al. (2020), Nurerk and Junden (2021), Preethi et al. (2021), Reina et al. (2022), Maciel (2022) and donné Kiatti et al. (2024)
Lipids	0.24–5.22	0.86–12.06	%	Sancho et al. (2015), Guedes‐Oliveira et al. (2016), Duarte et al. (2017), Kouassi et al. (2018), Singh et al. (2019), de Medeiros et al. (2020), Nurerk and Junden (2021), Amaral et al. (2021) and Portela (2022)
Ash	0.17–2	1.48–2.7	%	Ogunjobi and Ogunwolu (2010), Kuila et al. (2011), Sancho et al. (2015), Guedes‐Oliveira et al. (2016), Duarte et al. (2017), Kouassi et al. (2018), Singh et al. (2019), de Medeiros et al. (2020) and Nurerk and Junden (2021)
Total sugar	2.5–7.7	2.2–30.6	g/100 g	Santos et al. (2007), Uchoa et al. (2009), Guedes‐Oliveira et al. (2016), Singh et al. (2019) and Portela (2022)
Reducing sugar	6.84–7.25	13–58.69	g/100 g	Matias et al. (2005), Kuila et al. (2011), Sancho et al. (2015), Adegunwa et al. (2020), de Araujo Padilha et al. (2020) and Osei et al. (2024)
Crude fiber	11.79–12.5	11.03–79	g/100 g	Nagaraja et al. (2007), Guedes‐Oliveira et al. (2016), Batista et al. (2018), Singh et al. (2019), de Medeiros et al. (2020), Muniz et al. (2020), Nurerk and Junden (2021), Maciel (2022), Portela (2022) and Osei et al. (2024)
Soluble fiber	0.04–8.8	7.4–8.08	%	Duarte et al. (2017), de Medeiros et al. (2020), Nurerk and Junden (2021), Portela (2022) and Maciel (2022)
Insoluble fiber	0.96–91.92	27–39	%	Batista et al. (2018), Muniz et al. (2020), Nurerk and Junden (2021) and Portela (2022)

Up to 58.69 mg/100 g of reducing sugars have been reported by Kuila et al. (2011) using aqueous extraction techniques from CAP. In food applications, the reducing sugars (glucose and fructose) may be essential for consumer acceptability as they provide excellent solubility, sweet taste, and the ability to take part in Maillard reactions with amines to enhance flavours and colorus (BeMiller 2017).

CAP is also rich in indigestible carbohydrates, notably dietary fibre (soluble and insoluble). This presents a viable opportunity for the development of functional foods with CAP to deliver health benefits such as regulating glucose absorption and reducing risks of cardiovascular diseases and Type 2 diabetes (Akyereko et al. 2023; Aslam et al. 2024). CAP dietary fibre may provide prebiotic benefits like slow digestion and enhanced satiety (do Nascimento et al. 2021; Menezes et al. 2021). In a 24‐h in vitro fermentation with pooled human faecal inoculum study, freeze‐dried CAP increased beneficial *Bifidobacterium* and *Lactobacillus/Enterococcus*, while reducing *Bacteroides/Prevotella* and *Clostridium histolyticum* (Menezes et al. 2021). do Nascimento et al. (2021) confirmed the prebiotic potential of CAP by showing that CAP promoted *Bifidobacterium lactis* growth (8.8 Log CFU/mL) after 12 h of fermentation, with a slight pH reduction. These findings highlight CAP's potential as a functional food ingredient with significant prebiotic benefits for human health.

While CAP fibre may provide these health benefits highlighted, the bioaccessibility of nutrients in the formulated food product may be hampered due to insoluble fibre and phytic acid present in CAP. Phytic acid and fibre bind strongly to metallic cations like K, Mn, Mg, Ca, Fe, and Zn, forming mixed‐salt molecules called phytin, which reduces the bioavailability of micronutrients in humans (Moreira et al. 2013). A study showed that CAP contained higher copper levels than cashew apple juice (12.20 vs. 2.10 mg/L); however, its bioaccessibility was reportedly lower during an in vitro digestion experiment (de Lima et al. 2014). This reduced bioaccessibility was associated with phytic acid (0.25%) present in the CAP.

### 4.1 Minerals and Vitamin Composition

Okpanachi et al. (2015) reported that CAP contains Vitamin A (0.86–1.48 mg/100 mL) and E (0.11–0.14 mg/100 mL). Although the majority of vitamins are found in cashew apple juice, Table (2) shows that significant quantities of vitamins—particularly vitamin C— are present in CAP (20.3–901 and 2.0–352.8 mg/100 g dry and wet basis, respectively). It is fair to highlight that the processing methods employed in juice extraction and CAP processing may influence the vitamin C level in the resulting pomace. The vitamin C composition of CAP may contribute to CAP's antioxidant activity and may be useful in alleviating vitamin C deficiency and the treatment of associated syndromes (Aidoo et al. 2022).

**TABLE 2:** Mineral and vitamin composition of CAP.

Mineral (mg/g)	Wet CAP	Dry CAP	References
Phosphorus (P)	1.45	1.28–15.33	Santos et al. (2007), Sancho et al. (2015), Msoka et al. (2017), de Medeiros et al. (2020) and Preethi et al. (2021)
Potassium (K)	4.92–83.5	15.6–83.5	Sancho et al. (2015), Msoka et al. (2017), de Medeiros et al. (2020), Preethi et al. (2021) and Patra et al. (2021).
Sodium (Na)	0.54	0.25	Sancho et al. (2015) and Patra et al. (2021)
Zinc (Zn)	0.01–0.07	0.1–9.9	de Medeiros et al. (2020), Patra et al. (2021) and Reina et al. (2022)
Copper (Cu)	18.3	0.3–41.92	de Medeiros et al. (2020) and Preethi et al. (2021)
Boron (B)	—	1.14	Preethi et al. (2021)
Magnesium (Mg)	8.13	1.03–4.5	Podrigues et al. (2007), Sancho et al. (2015), de Medeiros et al. (2020) and Preethi et al. (2021)
Iron (Fe)	0.049	0.02–17.96	Podrigues et al. (2007), Sancho et al. (2015), de Medeiros et al. (2020) and Preethi et al. (2021)
Calcium (Ca)	0.99	7.4–26.1	Santos et al. (2007), Msoka et al. (2017), de Medeiros et al. (2020), Patra et al. (2021), Preethi et al. (2021) and Osei et al. (2024)
**Vitamins**			
Ascorbic acid	20.3–901	2.0–352.8 (mg/100 g)	de Medeiros et al. (2020), Preethi et al. (2021), Reina et al. (2022) and Osei et al. (2024)
Vitamin A	—	0.86–1.48 (mg/100 mL)	Okpanachi et al. (2015)
Vitamin E	—	0.11–0.14 (mg/100 mL)	Okpanachi et al. (2015)

CAP contains total ash content (1.48%–2.7%) and has been reported to be present in CAP (Table 1), signifying its richness in minerals (Nurerk and Junden 2021; Reina et al. 2022). CAP contains minerals including P, K, Mg, Ca, Na, Zn, and Fe (Costa et al. 2009). Based on the data presented in Table 2, potassium, copper, calcium, and iron are the most abundant minerals in CAP. Iron is essential for biological functions like oxygen transport and cellular respiration (Katsarou and Pantopoulos 2020). Calcium is crucial for improving bone health and lowering systolic blood pressure (Prentice et al. 2024; Voulgaridou et al. 2023) and potassium is vital for supporting muscle development and regulating blood pressure (de Freitas et al. 2023). Essentially, CAP may be a good source of these minerals for incorporation into the Dietary Approaches to Stop Hypertension (DASH) diet for hypertension management (Akyereko et al. 2023). The high levels of essential minerals in CAP may support strong immunity, proper fluid balance, nerve transmission, and muscle contraction (Gawankar et al. 2018). Micronutrient deficiencies, including iron and zinc, are prevalent in sub‐Saharan Africa (Haidar et al. 2003). Instead of allowing cashew apples (CA) and CAP to go to waste, they can be used to enrich diets and help address these deficiencies. To efficiently utilize the mineral richness in CAP for food product development, pretreatments and processing techniques such as soaking, fermentation, and addition of phytase may be used as a pretreatment method during CAP processing to obstruct Phytic acids from reducing mineral bioavailability. Optimizating formulations may also be considered to ensure the optimal inclusion of CAP fibre for improved mineral bioaccessibility.

### 4.2 Amino Acid Composition

The amino acid composition of cashew apple pomace (CAP), as presented in Table 3, highlights its potential as a valuable protein source. CAP contains essential and nonessential amino acids, which play crucial roles in physiological functions, growth, and metabolism (Apine and Jadhav 2019; Leite et al. 2021). Glutamic acid, leucine, aspartic acid, proline, alanine, lysine, glycine, phenylalanine, threonine, arginine, and serine constitute the major fraction of amino acids in CAP (Okpanachi et al. 2015). Certain amino acids can be decarboxylated by specific amino acid decarboxylases to produce amines that play important physiological roles. For example, tryptophan (Trp), when catalyzed by tryptophan hydroxylase (TPH), produces 5‐hydroxytryptamine (5‐HT), a neurotransmitter that also constricts peripheral blood vessels (Petroff 2002). Glutamic acid (Glu) decarboxylates to generate gamma‐aminobutyric acid (GABA), an inhibitory neurotransmitter that promotes a calming effect on the central nervous system (Jing et al. 2007). Histamine, produced from histidine (His) through histidine decarboxylase, is widely distributed in the body. It plays a key role in vasodilation and is released at wound and inflammation sites (Deng et al. 2007). Branched‐chain amino acids such as leucine promote tissue synthesis including skeletal muscle, heart, and brain (Li et al. 2009), and modulate insulin activity, aiding blood glucose regulation in sarcopenic patients and the elderly (Manders et al. 2012).

**TABLE 3:** Amino acid composition of cashew apple pomace.

Amino acid	Value (g/100 g)	Amino acid	Value (g/100 g)
Ala	1.99–2.28	Leu	2.79–3.11
Arg	1.47–1.90	Lys	1.84–2.22
Asp	3.32–3.97	Met	0.49–0.63
Cys	0.40–0.66	Phe	1.87–1.94
Glu	3.64–4.24	Pro	2.24–2.65
Gly	1.83–2.09	Ser	1.52–1.79
His	0.76–1.04	Thr	1.41–1.93
Ile	1.65–1.97	Tyr	1.11–1.27

*Note:* Adapted from Okpanachi et al. (2015).

### 4.3 Bioactive Compounds

Various bioactive compounds found in CAP including phenolic acids, flavonoids, tannins, and carotenoids are summarized in Table 4. These bioactive compounds are notable for preventing diseases and inflammations in the body (Wu et al. 2023), antioxidative and anticarcinogenic properties, cardioprotective properties, and other therapeutic functions (Mba et al. 2019; Prasertsri and Leelayuwat 2017). Phenolic compounds comprise a broad class defined by the presence of a phenol ring (Silva et al. 2023). As shown in Table 3, the total phenolic compound (TPC) is the highest since it includes all phenolic compounds in the sample, such as tannins, anthocyanins, flavonols, and other minor phenolics (Barroso et al. 2024). According to da Silva et al. (2014), CAP contains carotene (174 μg/100 g) and yellow flavonoids (44.91 mg/100 g) on a dry basis. CAP has tannins (313.00 mg CE/100 g), flavonols (109.03 mg QE/100 g), carotenoids (67.20 μg β‐carotene/g), anthocyanins (36.05 mg cyanidin‐3‐glycosides/100 g), and TPC (1975.64 mg GAE/100 g) (Andrade et al. 2015). CAP is rich in carotenoids, mainly because the pigments are embedded in the tissues (de Lima et al. 2014).

**TABLE 4:** Phenolic compounds in wet and dried CAP.

Bioactives	Wet CAP	Dried CAP	Unit	References
Total phenolics	49.8–97.44	20–1975.64	mg GAE/100 g	Sancho et al. (2015), Sucupira et al. (2020), de Medeiros et al. (2020), Preethi et al. (2021) Reina et al. (2022), Osei et al. (2024) and Fonteles et al. (2017)
Phenolic acids	—	150–303.8	Mg CAE/100 g	Osei et al. (2024)
Tannins	—	0.07–313	Mg CE/100 g	Nagaraja et al. (2007), Msoka et al. (2017), Sucupira et al. (2020), de Medeiros et al. (2020), Reina et al. (2022) and Andrade et al. (2015)
Carotenoids	5.27–5.24	1.71–67.20	mg/100 g	Sucupira et al. (2020), de Medeiros et al. (2020), Reina et al. (2022) and Osei et al. (2024)
Flavonoids	—	4.53–233.57	mg CE/100 g	de Medeiros et al. (2020), Reina et al. (2022) and Osei et al. (2024).
Anthocyanins	—	2.46–36.05	mg CE/100 g	Sancho et al. (2015) and Reina et al. (2022).
Proanthocyanidin	—	62.2	mg PAE/100 g	de Medeiros et al. (2020)
Antioxidant activity	—	10.63–17.8 405	mg AAE/g TEs/g	Preethi et al. (2021), Reina et al. (2022) and Sancho et al. (2015).

Abbreviations: — symbol means information is not available; AAE, ascorbic acid equivalent; CAE, caffeic acid equivalent; CAP, cashew apple pomace; CE, catechin equivalents; GAE, gallic acid equivalent; PAE, proanthocyanidin equivalents; QE, Quercetin equivalent; TEs/g, Trolox equivalents per gram.

Tannins are polyphenols classified as antinutrients due to their negative effect on nutrient absorption and bioavailability (Farha et al. 2020; Popova and Mihaylova 2019). Tannins in CAP, known for their astringency and bitterness, can bind or precipitate proteins and other organic compounds, such as amino acids, reducing their digestibility and bioavailability (Thakur et al. 2019). Despite their negative effects, tannins possess beneficial properties, including antioxidant, antitumor, anti‐inflammatory, anthelmintic, and antimicrobial activities (Sharma et al. 2021). Carotenoids, as precursors to vitamin A, are crucial for ocular and immune health and play a role in lowering the risk of various degenerative diseases, including cancer, cardiovascular conditions, and cataracts (Bohn et al. 2021; Patra et al. 2022), whereas anthocyanins may help protect against cardiovascular diseases (Mohammed and Khan 2022). The high flavonoid content of CAP has also been linked to weight loss (Akyereko et al. 2023). In mice fed a high‐fat diet, CAP was shown to control appetite, prevent elevated blood sugar, insulin resistance, blood lipid levels, and reduce diet‐related liver damage. These effects were attributed to the high fibre and bioactive compounds in CAP, which are known to enhance satiety and support the metabolism of lipids and glucose (Carvalho et al. 2019).

### 4.4 Phenolic Compounds

**FIGURE 3:**
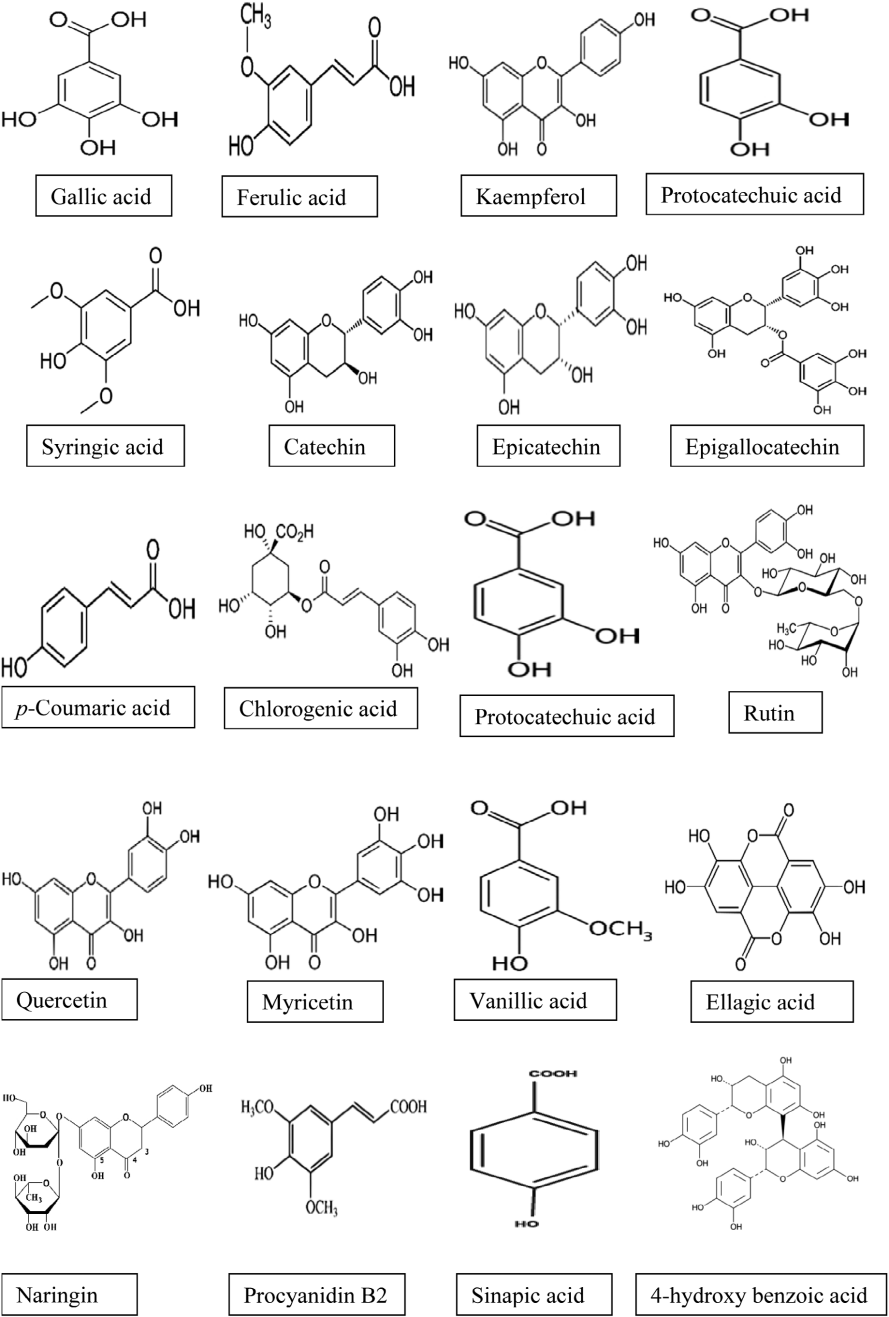
Structure of phenolic compounds in cashew apple pomace.

Several phenolic compounds have been identified and quantified in CAP (Table 5). Polyphenolic compounds, such as flavonoids (anthocyanins, myricetin, quercetin, kaempferol), tannins, and phenolic acids (caffeic acid, coumaric acid, ferulic acid, and gallic acid) are prominent constituents of CAP. Figure 3 shows the chemical structure of various phenolic compounds found in CAP. HPLC analysis of the CAP methanol/water (70:30 v/v) extract using a UV/VIS SPD‐20^0^ detector identified several phenolic compounds. Naringin, Protocatechuic acid, and Gallic acid emerged as the most prevalent phenolics (donné Kiatti et al. 2024). With enzymatic extraction followed by HPLC‐LC–MS/MS analysis, myricetin, vanillic acid, and gallic acid were identified and quantified as the major phenolic compounds in CAP (Table 4) (de Freitas et al. 2023). The high flavonoid content of CAP has also been linked to weight loss (Akyereko et al. 2023). Derivatives of myricetin and quercetin, contained in cashew apple extract, also present in CAP, effectively reduced fat accumulation and insulin resistance. Moreover, cashew apples reportedly reduce body weight gain, fat storage, hyperglycemia, hyperinsulinemia, and insulin resistance in the obese mice (Gutiérrez‐Paz et al. 2024). Additionally, myricetin, which is abundant in CAP, has shown anti‐inflammatory activity by suppressing proinflammatory mediators (Cho et al. 2016), cardiovascular effects (Zhang et al. 2018), and anticancer activity (Ci et al. 2018). CAP extract enriched with carotenoids and anacardic acids (100 mg/kg, p.o.) showed improved gastric mucosa and antioxidant levels in aspirin‐induced gastric lesions in rats. The extract increased glutathione and IL‐10 while reducing IL‐1β, inflammatory proteins, and myeloperoxidase activity, indicating a gastroprotective and anti‐inflammatory effect of CAP (Menezes et al. 2021). The findings suggest that CAP can serve as a functional food ingredient for preventing or managing gastric ulcers while mitigating oxidative and inflammatory damage. From our recent work, CAP showed potent antimicrobial activity against bacteria like *Yersinia enterocolitica* CCM 5671, *Escherichia coli* CCM 3954, *Salmonella enterica* subsp. enterica CCM 3807, *Candida krusei* CCM 8271, *Candida albicans* CCM 8186, *Candida parapsilosis* CCM 8260, and *Candida tropicalis* CCM 8223. This finding may be attributed to the phenolic compounds in CAP, such as ferulic acids. Based on this finding, CAP application in food products may protect consumers against bacteria and yeast infections (Osei et al. 2024).

**TABLE 5:** Phenolic compounds in cashew apple pomace.

Phenolic compound	de Freitas et al. (2023)	donné Kiatti et al. (2024)
	mg/kg	μg/g
Catechin	5.46–6.75	—
Chlorogenic acid	4.82–4.89	8.80–30.66
Ellagic acid	4.69–5.52	97.00–109.1
Epicatechin	5.71–6.88	—
Epigallocatechin	5.01–5.03	—
Ferulic acid	3.50–3.70	10.34–44.28^a^
Gallic acid	11.34–20.25	103.63–140.86
4‐Hydroxy benzoic acid	—	10.52–25.69
Myricetin	43.03–44.26	—
Naringin	—	469.8–1477
Protocatechuic acid	3.26–8.09	378.9–696.1
Procyanidin B2	—	22.74–50.38
*p*‐Coumaric acid	7.96–12.27	8.00–26.97
Quercetin	5.21–5.30	43.93
Rutin	6.66–13.55	—
Syringic acid	4.52–5.02	—
Sinapic acid	—	13.12–34.34
Vanillic acid	30.96–32.32	—
*trans*‐cinnamic acid	—	37.05–81.17

Abbreviations: — symbol means information is not available.

^a^ *trans*‐ferulic acid.

## 5 Applications of Cashew Apple Pomace in Food Products

**FIGURE 4:**
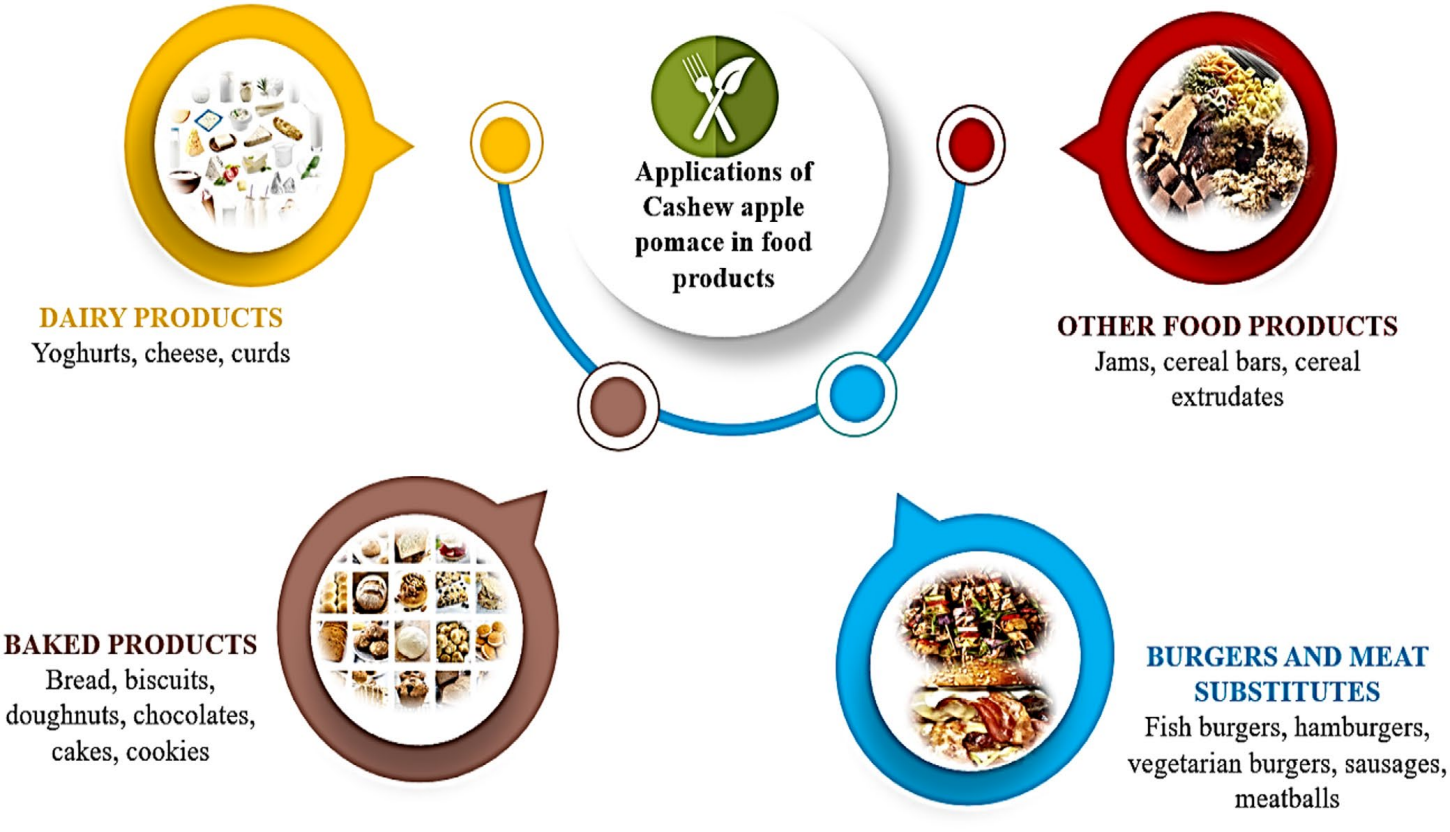
Applications of cashew apple pomace in food products.

CAP can be employed in developing various value‐added prebiotics, nutraceuticals, and functional foods (Chen et al. 2023). CAP can be incorporated into food products in the raw, powder, or extract form. Figure 4 highlights products such as dairy, baked goods, meat and meat substitutes, cereal extrudates, and other food products with CAP as an ingredient.

### 5.1 Dairy Products

CAP as a source of bioactive compounds was incorporated into yogurt during preparation. Sensory analysis revealed that adding 10% and 20% CAP extract produced a product that was acceptable to consumers (Mendes et al. 2019). The production of probiotic fermented milk using CAPP as a functional substrate was studied. It was discovered that CAPP used as a feeding matrix for *Lacticaseibacillus paracasei* subsp. *paracasei* F19 (F19) and *Streptococcus thermophilus* ST‐M6 (ST‐M6) gave fermented milk favorable characteristics. The phenolic and antioxidant activity of the fermented milk mixture was considerably enhanced by CAPP during storage (Herkenhoff et al. 2023). In a static in vitro gastrointestinal simulation assay, CAP effectively protected probiotic strains and the starter, indicating its suitability for producing probiotic fermented milk (Herkenhoff et al. 2023).

### 5.2 Burgers and Meat Substitutes

Marques (2018) used CAPP in place of mechanically separated fish at 0%, 5.88%, 11.76%, and 17.64% during the formulation of fish burgers. The carotenoid content and antioxidant activity of the CAP‐based burger increased while fat and protein content decreased. Based on the sensory results, the CAP‐based burger and control burger had a purchased attention (score of 4.25 vs. 4.56) and an overall impression (of 6.93 vs. 7.81), respectively (Marques 2018). Lima (2008) studied the physicochemical and sensory characteristics of a vegetarian hamburger made from CAP. Four commercial burgers (beef, soy, vegetable, and vegetable with chicken taste) were compared to the cashew‐enriched burger produced by adding CAP at 89% ratio. The cashew burger had less protein (5.75%), fat (7.9%), moisture (49.47%), ash (2.89%), and pH (4.75) than all the control burger. The panelists gave the CAP burger a lower sensory score (5.9) than the commercial burgers (6.5). Pinho et al. (2011) investigated the substitution of meat with CAPP in low‐fat hamburger preparation, ranging from 0% to 14.37%. Higher CAPP levels increased total carbohydrates and dietary fibre while reducing moisture, protein, and lipid content by 35%. Total dietary fibre content ranged from 0% to 7.66%, mainly from insoluble fibre. Sensory results showed that up to 10.70% CAPP substitution had no significant impact on flavour. The researchers suggested that the partial replacement of meat with CAPP in hamburgers could create products that are high in dietary fibre and low in fat (Pinho et al. 2011). Cashew burgers were made by de Oliveira Rosa and Lobato (2020) using juice (F1) and fibre (F2). The “colour attribute” was linked to an average of 6.83 (F1) and 6.72 (F2), the “flavour attribute” to 6.97 (F1) and 6.87 (F2), and the “texture attribute” to 6.35 (F1) and 6.56 (F2). F1 and F2 had global averages of 7.27 and 7.12, respectively, and “liked it very” and “liked it very much” were the purchase intentions. As a result, there was a slight preference for cashew juice‐based hamburgers compared to fibre‐based ones. Lima et al. (2018) studied a vegan burger formulation that included 27% CAP from two treatments (enzyme‐treated and untreated). Enzyme‐treated CAP had lower moisture (70.04%), ash (2.21%), proteins (5.77%), lipids (1.01%), and carbohydrates (20.97%) compared to untreated CAP. During storage at −18°C, ascorbic acid, pH, and adhesiveness significantly decreased, while acidity, hardness, and cohesiveness increased. The enzyme‐treated CAP showed no sensory differences, with an acceptability score of 7.6 and a purchase intention of 3.9, which remained unchanged during frozen storage.

Hamburger was developed with CAPP at inclusion levels of 20% (F1), 30% (F2), and 50% (F3). When compared with regular hamburgers, the product had a reduced fat level and higher fibre content, which indicated its superior nutritional value. Along with a high zinc concentration, it also had a rich source of protein, vitamin C, and fixed mineral residue. A higher acceptability was found for the hamburger with 30% CAPP (Barros et al. 2012). The washed CAPP was substituted for vegetable fat in chicken patties at 60%, 70%, 80%, and 90% (3%–4.5% of total ingredients). At 4.5% CAPP, lipid content decreased from 7.62% to 4.40%, and energy value dropped from 5.49 kcal/g to 4.95. The total carbohydrates increased from 6.61% to 9.92%, and cooking output also increased from 84.89% to 88.39%. Substitutions up to 90% were well accepted by the panelists (Guedes‐Oliveira et al. 2016).

### 5.3 Bakery Products

CAP additions of 5%, 10%, and 15% were used to make enhanced cookies. The cookies with the addition of 10% CAP demonstrated a high degree of flavour acceptance (Matias et al. 2005). In contrast to the findings of de Araújo et al. (2021), Akubor (2016) discovered that the biscuit made with 10% CAPP had a higher carbohydrate content than the biscuit made with 100% wheat flour. The CAPP replaced wheat flour at 0%, 5%, 10%, 15%, and 20% for the cookie formulation. The colour of the final products deepened as CAPP incorporation increased, whereas the amount of dietary fibre increased. The panelists preferred cookies with 15% CAP above the control (Uchoa et al. 2009). CAPP replaced wheat flour in cookies at 0%, 10%, and 15%. Higher CAPP levels increased acidity, moisture, ash, fibre, protein, and fat, while pH and carbohydrates decreased. The cookies with 10% CAPP achieved the highest score of 6.8, and improved texture (7.2) and fragrance (7.08) scores (de Araújo et al. 2021). A reduced total carbohydrate content was observed when CAPP was added to wheat for cookie production (Ebere et al. 2015). In preparing biscuits, CAPP was used replcaed of wheat flour at levels of 0%, 5%, 10%, 20%, 30%, 40%, 50%, and 100%. Biscuits containing 10% CAPP had higher levels of ash (1.7%), fibre (3.2%), and carbohydrates (67.6%) but lower levels of protein (4.5%) and moisture. With CAPP substitution, there was a decrease in weight, height, and spread ratio. As the CAPP addition increased, the biscuit darkened, and low consumer acceptability was recorded (Akubor 2016). After 6 months of storage, chocolate incorporated with CAPP maintained very low bacteria, with yeast and fungus, suggesting improved storage stability (Sobhana et al. 2015). In place of wheat flour, solar‐dried CAPP was used at percentages of 5%, 10%, and 20% for buns and 10% for cakes. The study found no discernible flavour difference between buns with 5% and 10% CAPP. The cake received a rating of 7.6, and the addition of CAPP did not alter the cake's acceptance (Moura et al. 2015). The researchers found that fermentation or yeast negatively impacted the incorporation of higher CAPP amounts, requiring longer baking times and higher temperatures (Moura et al. 2015).

### 5.4 Other Food Products

Nguyen et al. (2023) investigated the effects of varying CAPP concentrations on pasta quality. Increasing CAPP from 0% to 20% enhanced total phenolic content and dietary fibre by 4.1 and 11.8 times, respectively, while antioxidant power and radical scavenging activity improved by 18.2 and 28.6 times. However, higher CAPP levels reduced the pasta's overall acceptability, textural qualities, and cooking quality. The pasta with 10% CAPP was considered a high‐fibre product with acceptable sensory quality (Nguyen et al. 2023). Sucupira et al. (2020) examined bioactives in handcrafted and industrial cashew apple fibre (CAF) used to produce CAF meatballs. The bioactive compounds present in both types of fibre were abundant, with artisanal fibre exhibiting high levels of ascorbic acid (147.8 mg/100 g) and industrial fibre having notable quantities of carotenoids (1.87 mg/100 g). Cereal extrudates were enriched by adding CAPP to rice flour at 0%–25% (Preethi et al. 2021). This addition increased protein, starch, ash, and total mineral levels, except for boron and phosphorus. The extrudates with CAPP showed higher levels of phenolics (4.00 mg GAE/g), flavonoids (0.35 mg CE/g), and total antioxidant activity (5.34 mg AAE/g). Sensory scoring indicated that CAPP could be added to snacks at 5%–15% and to fish and animal feed extrudates up to 20% (Preethi et al. 2021). Jam prepared with CAPP at varying concentrations of cashew juice (0%, 5%, 10%, and 15%), along with water, sugar, pectin, and salt. Results indicated that the colour of the jam darkened with the addition of cashew juice. The highest sensory scores were observed for the CAPP jam containing 5% cashew juice, scoring 7.60, compared to 6.47 for the control (Nurerk and Junden 2021).

Notably, most studies focused on the nutritional composition of CAP‐enriched products without studying the bioavailability of the nutrients and bioactive compounds. Additionally, extensive nutritional profiling and bioactivity of CAP‐incorporated products is presently limited. This warrants further research using in vitro and in vivo study models to validate the health benefits of products. Moreover, in most studies, undesirable sensory changes in CAP‐enriched products were observed to impact consumer acceptance. Strategies like formulation optimization, flavour masking, texture modification, hydration, and processing techniques such as fermentation and extrusion may be employed to enhance product quality. These approaches may help create nutritious and appealing CAP‐based food products.

## 6 Conclusion and Future Perspectives

CAP is rich in nutrients, phenolic compounds, and dietary fibre that offer various health benefits, including antioxidant, antimicrobial, antidiabetic, and anti‐inflammatory properties. The CAP provides an opportunity to address environmental and nutritional concerns by transforming waste into valuable ingredients for functional foods. While CAP's nutritional potential is well‐documented, further research is needed to address lapses. There is a need for studies on the health benefits of food products incorporated with CAP in in vitro and in vivo studies. Addressing these gaps requires human and animal trials, standard methodologies, and exploration of innovative processing techniques. Comprehensive characterization of phenolic compounds and amino acids present in CAP varieties is needed as present information is scanty. Studies that focus on reducing undesirable sensory qualities are essential for higher consumer acceptance and product quality. Expanding research in this direction could unlock CAP's full potential, not only as a sustainable food source but as a key ingredient in health‐promoting products. The potential of CAP in the functional food sector is highly promising. With ongoing research and technological advancements, CAP could emerge as a key ingredient for creating nutrient‐rich, health‐boosting products that align with consumer demands while supporting sustainability within the food industry.

## Author Contributions

**Emmanuel Duah Osei:** writing – original draft (lead), writing – review and editing (equal). **Anthony Amotoe‐Bondzie:** writing – original draft (supporting), writing – review and editing (equal). **Abigail Ataa Pokuah:** validation (lead). **Wisdom Sambian Laar:** validation (supporting). **Newlove Akowuah Afoakwah:** conceptualization (equal), writing – review and editing (equal). **Eva Ivanišová:** conceptualization (equal), supervision (equal).

## Ethics Statement

The authors have nothing to report.

## Consent

The authors have nothing to report.

## Conflicts of Interest

The authors declare no conflicts of interest.

## Data Availability

The authors have nothing to report.
